# Specific recognition and inhibition of Ewing tumour growth by antigen-specific allo-restricted cytotoxic T cells

**DOI:** 10.1038/bjc.2011.54

**Published:** 2011-03-15

**Authors:** U Thiel, S Pirson, C Müller-Spahn, H Conrad, D H Busch, H Bernhard, S Burdach, G H S Richter

**Affiliations:** 1 Laboratory for Functional Genomics and Transplantation Biology, Children's Cancer Research Center and Department of Pediatrics, 81664 München, Germany; 2 III. Medizinische Klinik and Poliklinik (Hematology/Oncology), 81664 München, Germany; 3 Institute of Microbiology, Immunology and Hygiene, Roman Herzog Comprehensive Cancer Research Center and Klinikum rechts der Isar, Technische Universität München, 81664 München, Germany; 4 Department of Hematology/Oncology, Klinikum Darmstadt, 64283 Darmstadt, Germany

**Keywords:** Ewing tumour, cytotoxic CD8^+^ T cells, immunotherapy, adoptive transfer, multimer technology

## Abstract

**Background:**

The development of a successful immunotherapy is hampered by an ineffective T-cell repertoire against tumour antigens and the inability of the patient's immune system to overcome tolerance-inducing mechanisms. Here, we test the specific recognition and lytical potential of allo-restricted CD8^+^ T cells against Ewing tumour (ET) associated antigens Enhancer of Zeste, Drosophila Homolog 2 (EZH2), and Chondromodulin-I (CHM1) identified through previous microarray analysis.

**Methods:**

Following repetitive CHM1^319^ (VIMPCSWWV) and EZH2^666^ (YMCSFLFNL) peptide-driven stimulations with HLA-A^*^0201^+^ dendritic cells (DC), allo-restricted HLA-A^*^0201^−^ CD8^+^ T cells were stained with HLA-A^*^0201/peptide multimers, sorted and expanded by limiting dilution.

**Results:**

Expanded T cells specifically recognised peptide-pulsed target cells or antigen-transfected cells in the context of HLA-A^*^0201 and killed HLA-A^*^0201^+^ ET lines expressing the antigen while HLA-A^*^0201^–^ ET lines were not affected. Furthermore, adoptively transferred T cells caused significant ET growth delay in Rag2^−/−^*γ*_C_^−/−^ mice. Within this context, we identified the CHM1^319^ peptide as a new candidate target antigen for ET immunotherapy.

**Conclusion:**

These results clearly identify the ET-derived antigens, EZH2^666^ and CHM1^319^, as suitable targets for protective allo-restricted human CD8^+^ T-cell responses against non-immunogenic ET and may benefit new therapeutic strategies in ET patients treated with allogeneic stem cell transplantation.

T-cell based tumour immunology suffers from a principal dilemma: tumour-derived peptides are frequently self-antigens associated with MHC class I molecules. Moreover, T cells with high affinity for such antigens undergo negative selection and peripheral tolerance mechanisms diminish their number or eliminate self-peptide specific cytotoxic T cells. Nevertheless, as the T-cell repertoire has not been educated to ignore self antigens presented by foreign MHC molecules, allo-restricted T cells may represent a comprehensive repository for tumour-specific T cells (Felix and Allen, 2007).

Allogeneic stem cell transplantation (SCT) is an established treatment for leukaemia where donor T cells induce a graft-*vs*-leukaemia response that can eradicate residual malignant cells (Kolb *et al*, 1995), and is now being explored as a treatment for a variety of other haematologic and non-haematologic malignancies (Childs *et al*, 2000). For malignant peripheral neuroectodermal tumours (Ewing tumour, ET), patients with vast bone affection and poor prognosis, allogeneic SCT represents a therapy option (Burdach *et al*, 2000, 2009). Koscielniak *et al* (2005) and Lucas *et al* (2008) reported tumour regression in ET patients with advanced disease immediately after allogeneic SCT. This possible graft-*vs*-ET effect, however, may be associated with a pronounced toxicity potential of a graft-*vs*-host response in this therapeutic approach.

During the past years, methods emerged to identify, isolate, and expand tumour peptide-specific allo-restricted T cells *ex vivo* (Moris *et al*, 2001; Dutoit *et al*, 2002; Mutis *et al*, 2002; Amrolia *et al*, 2003; Whitelegg *et al*, 2005; Schuster *et al*, 2007), anticipating their potential use for adoptive immunotherapy (Rosenberg *et al*, 2008) for example, to replace common donor lymphocyte infusion (DLI) with tumour-specific allo-restricted T cells. We describe here, an HLA-A^*^0201-multimer approach using peptides derived from genes identified to be overexpressed in ET by microarray analysis (Staege *et al*, 2004). These peptide/MHC multimers enabled the selection of allo-restricted tumour-antigen specific T cells from an allo-reactive T-cell pool. Such T cells were peptide-specific and cytotoxic against ET cells with the appropriate HLA-expression and significantly delayed tumour growth after adoptive transfer in a xenograft mouse model.

## Materials and methods

### Cell lines

MHHES1, SK-ES1, SK-N-MC, TC71 (ET cell lines), CHP126, MHHNB11, SH-SY5Y, SIMA (neuroblastoma cells), and NALM6, 697, cALL2 (paediatric human B-cell precursor leukaemic lines) were obtained from the German Collection of Microorganisms and Cell Cultures (DSMZ; Braunschweig, Germany). The HLA-A^*^0201^+^ melanoma cell line SK-MEL29 was provided by L Old (Memorial Sloan-Kettering Cancer Institute, New York, NY, USA). A673 (ET cells) and Cos-7 (Simian SV40-transformed fibroblasts) were obtained from ATCC (LGC Standards GmbH, Wesel, Germany), the TAP-defective HLA^*^A0201^+^ T2 cell line (LCL somatic cell hybrid) was from P Cresswell (Yale University School of Medicine, New Haven, CT, USA). The HLA-A^*^0201^–^ erythroid leukaemia cell line K562 was a gift from A Knuth and E Jäger (Krankenhaus Nordwest, Frankfurt, Germany). HLA-A^*^0201^–^ SBSR-AKS ET cells were described previously (Richter *et al*, 2009). All cell lines are routinely tested for purity (e.g., translocation product, surface antigen or HLA-phenotype) and mycoplasma contamination. Lymphoblastoid cell lines (LCL) were generated by EBV transformation of peripheral blood B cells from HLA-A^*^0201^+^ healthy donors by use of a mini-EBV plasmid (Moosmann *et al*, 2002). The supernatant was provided by Josef Mautner and Andreas Moosmann, Helmholtz-Zentrum München. Tumour cell lines including K562 cells were cultured in RPMI 1640 or DMEM (only Cos-7 and SK-Mel29; Life Technologies, Paisley, Scotland) supplemented with 10% foetal calf serum (FCS, Biochrom, Berlin, Germany), 100 U ml^−1^ penicillin, 100 *μ*g ml^−1^ streptomycin, and 2 mM glutamine (all from Life Technologies). RPMI 1640 medium for LCL and T2 cells was supplemented with 10% human AB serum, 2 mM L-glutamine, 1 mM Na-pyruvate, non-essential amino acids, and 50 *μ*g ml^−1^ gentamycine (all from Life Technologies).

### Isolation of PBMC

Peripheral blood mononuclear cells (PBMCs) were isolated from human peripheral blood samples of healthy donors (obtained with IRB approval and informed consent from the DRK-Blutspendedienst Baden-Württemberg-Hessen in Ulm, Germany) by centrifugation over Ficoll-Paque (GE Healthcare, Freiburg, Germany) according to the supplier's recommendations.

### Generation of dendritic cells (DCs)

CD14^+^ cells were isolated from PBMCs with anti-human CD14 magnetic particles (BD Biosciences, Heidelberg, Germany) according to the manufacturer's instructions. Purity of cells was confirmed by flow cytometry on a FACS Calibur (BD Biosciences).

CD14^+^ monocytes were cultured in X-Vivo15 (Biowhittaker/Cambrex Bio Science Verviers, Apen, Germany)/1% AB serum (Biowhittaker/Cambrex) with 1000 IU ml^−1^ IL-4 (R&D Systems, Wiesbaden, Germany) and 800 IU ml^−1^ GM-CSF (Leukine sargramostim, Bayer Health Care, Leverkusen, Germany) at a concentration of 3 × 10^5^ ml^−1^ with 25–30 ml per 75 cm^2^ cell culture flask (TPP, Trasadingen, Switzerland) at 37 °C and 5% CO_2_. On day 3, cytokines were replaced. On day 6 of culture, DC maturation was induced by adding a cytokine cocktail consisting of 10 ng ml^−1^ TNF*α*, 10 ng ml^−1^ IL-1*β*, 1000 IU ml^−1^ IL-6 (R&D Systems), and 1 *μ*g ml^−1^ PGE_2_ (Cayman Europe, Tallin, Estonia). On culture day 8 and 9, cells displayed a mature phenotype as evidenced by flow cytometry. DCs were considered mature when positive for CD86, CD83, and HLA-DR.

### Isolation of CD8^+^ T cells

Untouched CD8^+^ T cells were purified from human HLA-A^*^0201^–^ PBMCs by negative isolation technique using a cocktail of biotin-conjugated non-CD8 monoclonal antibodies and anti-biotin micro beads followed by depletion of magnetically labelled cells on LS columns (all from Miltenyi Biotec, Bergisch Gladbach, Germany). Purity of isolated CD8^+^ T cells was confirmed by flow cytometry.

### *In vitro* priming

Mature DCs were resuspended in T-cell medium (AIM-V supplemented with 5% human AB serum, 2 mM L-glutamine, and 50 *μ*g ml^−1^ gentamycine) and pulsed with selected peptides at a concentration of 30–50 μM in the presence of 20 *μ*g ml^−1^ *β*_2_M (Sigma, Taufkirchen, Germany) for 4 h at 37 °C and 5% CO_2_ washed and were then irradiated at 35 Gy, and used for T-cell priming immediately or stored in liquid nitrogen for subsequent experiments. CD8^+^ T cells from an HLA-A^*^0201^−^ donor were stimulated with allogeneic HLA-A^*^0201^+^ DCs in 200 *μ*l of T-cell medium in a stimulator to responder rate of 1 : 20 (5 × 10^3^ DCs per well : 10^5^ CD8^+^ T cells per well). For priming, T cells and DCs were co-cultured with 10 ng ml^−1^ rhIL-12 and 1000 U ml^−1^ rhIL-6 and after 1 week were restimulated with the same number of loaded DCs in the presence of 5 ng ml^−1^ rhIL-7 and 100 U ml^−1^ rhIL-2.

### Multimer-staining and cell sorting

Two weeks after the beginning of *in vitro* priming all activated T cells were pooled and stained with a specific peptide/HLA-A^*^0201-Pentamer-PE (Proimmune, Oxford, UK) and counterstained with an anti-human CD8-FITC mAb (BD Biosciences) for cell sorting. Isotype IgG mAb and irrelevant peptide/HLA-A^*^0201-Pentamer-PE served as a control. Cell sorting was executed on a FACS Aria (BD Biosciences).

### V*β* analysis of T-cell receptor repertoire

To determine the status of clonality of T-cell clones, the IOTest Beta Mark Kit (Beckman Coulter, Brea, CA, USA) was used. This kit is designed for flow cytometric determination of the T-cell receptor (TCR) V*β* repertoire of human T lymphocytes and allows testing for 24 different V*β* specificities that cover about 70% of the normal human TCR V*β* repertoire.

### Limiting dilution

After purifying peptide-specific T cells through peptide/HLA-A^*^0201-multimer-mediated cell sorting, isolated T cells were expanded using limiting dilution. Expansion was conducted in round-bottom 96-well plates in 200 *μ*l T-cell medium supplemented with anti-CD3 (30 ng ml^−1^), rhIL-2 (50 IU ml^−1^), rhIL-15 (2 ng ml^−1^), irradiated LCL; 1 × 10^5^ per well and irradiated PBMCs pooled from three different healthy donors (5 × 10^4^ per well) as feeder cells as previously described (Parker *et al*, 1994). Cytokines and 100 *μ*l medium/well were replaced after 1 week. Expanded T cells were further characterized in ELISpot assays.

### ELISpot-assay

The 96-well mixed cellulose ester plates (MultiScreen-HA Filter Plate, 0.45 *μ*m, Millipore, Eschborn, Germany) were coated overnight at 4 °C with 50 *μ*l per well of capture antibody solution (all Mabtech, Hamburg, Germany, Supplementary Table SII) in PBS. Plates were then washed four times with PBS and subsequently blocked with 150 *μ*l per well of TCM for 1 h at 37 °C. When peptide-loaded T2 cells were used, they were pre-incubated with 30–50 μM peptide for at least 2 h at 37 °C. When ET cells were used, they were pre-incubated with 100 U ml^−1^ IFN-*γ* 48 h before use in the assay. After blocking, the T cells to be investigated were either adjusted at a concentration of 2 × 10^6^ cells ml^−1^ in TCM and 50 *μ*l of serial dilutions (Granzyme B) or 50 *μ*l containing 1000 T cells (IFN-*γ*) were plated into the wells and incubated for 30 min at 37 °C. The target cells were washed, resuspended in TCM and 50 *μ*l per well allocated per well containing 20 000 cells. For HLA-A^*^0201 blocking of A673, the HLA class I (W6/32) specific antibody (Abcam, Cambridge, UK) was added to the wells at a concentration of 10 *μ*g ml^−1^. Peripheral blood mononuclear cells (PBMCs) isolated from an HLA-A^*^0201^+^ healthy donor were either stimulated with 3 *μ*g ml^−1^ OKT3 for 48 h or left untreated. Before application to the assay, cells were irradiated with 30 Gy and washed thrice with PBS. The plates were then incubated for 20 h at 37 °C. Subsequently, the plates were washed six times with PBS/0.05% Tween 20 (Sigma). Then wells were incubated for 2 h at 37 °C with 200 *μ*l of biotinylated secondary antibody (all Mabtech, Supplementary Table S2) diluted in PBS/0.5% BSA. The plates were washed six times with PBS/0.05% Tween 20. A volume of 200 *μ*l per well of Streptavidin-HRP (Mabtech) diluted 1/1000 was added and plates were incubated for 1 h at RT. After three washes with PBS/0.05% Tween 20 followed by three final washes with PBS, 100 *μ*l of 3-Amino-9-ethyl-carbazole solution (Sigma) was added and incubated for 4–8 min. Colour-development was stopped by washing under running tap water. Spots in dried plates were counted on an AID-ELIRIFL04 ELISpot reader (Autoimmun Diagnostika, Strassberg, Germany).

### Tumour challenge and adoptive T-cell transfer in Rag2^−/−^*γ*_C_^−/−^mice

Immunodeficient Rag2^−/−^*γ*_C_^−/−^ mice on a BALB/c background were obtained from the Central Institute for Experimental Animals (Kawasaki, Japan). Mice were bred and maintained in our animal facility under pathogen-free conditions in accordance with the institutional guidelines and approval by local authorities. Each mouse was challenged by s.c. intra-inguinal injection of 2 × 10^6^ A673 ET cells and monitored for tumour growth. Three days after tumour challenge, each mouse received either 2 × 10^6^ EZH2-15 (*n*=7) or 2 × 10^6^ CHM1-6 (*n*=6) T cells intravenously or were left untreated (control group, *n*=8). At 17 days after tumour challenge, mice were killed and analysed for tumour weight.

## Results

### Histone methyltransferase EZH2 and chondromodulin-I are strongly upregulated in Ewing tumours

**Figure 1:**
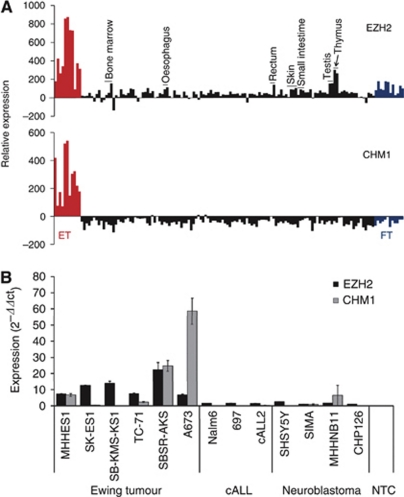
Antigen-specific expression profile (gray). (**A**) Expression profile of EZH2 and CHM1 in Ewing tumours (ET, red) in comparison to normal (black), and foetal tissue (FT, gray). Ewing tumour, FT and normal tissue samples were analyzed using EOS-Hu01 microarrays (Staege *et al*, 2004). (**B**) Expression of EZH2 and CHM1 was evaluated by real-time RT–PCR in different paediatric tumour cell lines. Error bars represent s.d. of triplicate experiments. Abbreviation: NTC=non-template control.

The EWS-FLI1 fusion protein, which is pathognomonic in 85% of ET, represents an ideal immunological target in search of immunogenic peptides for T-cell based therapy. However, we were not able to validate any peptide from this fusion region as a good binder to, for example, HLA-A^*^0201 (Meyer-Wentrup *et al*, 2005). Therefore, we reinforced our endeavours to identify cytotoxic T-cell epitopes of other antigens that are specifically expressed in ET. In a previous microarray analysis, we recognised the histone (lysine) methyl-transferase Enhancer of Zeste, Drosophila, Homolog 2 (EZH2) and Chondromodulin-I (CHM1) as strongly upregulated genes in ET (Staege *et al*, 2004) and demonstrated that EZH2 has a critical role in ET pathology by determining the oncogenicity and stem cell phenotype of this tumour (Richter *et al*, 2009). As shown in Figure 1A, CHM1 expression was not observed in any normal tissue analyzed, whereas EZH2 is expressed ubiquitously at low levels, with elevated levels in bone marrow, rectum, testis, and thymus. In addition, real-time RT–PCR demonstrated that other childhood malignancies including common acute lymphoblastic leukaemia (cALL) and neuroblastoma showed a significantly lower or no expression of CHM1 and EZH2, respectively (Figure 1B).

### Selection of HLA-A^*^0201-restricted peptides derived from ET antigens

HLA-A^*^0201 epitope binding analyses and presumed proteasomal cleavage prediction were performed by use of SYFPEITHI (Rammensee *et al*, 1999), BIMAS (Parker *et al*, 1994), and NetCTL (Larsen *et al*, 2005) algorithms (see Supplementary Information). Selected peptides and their scores are shown in Supplementary Table SI. Synthesised peptides were validated for binding to HLA-A^*^0201 onto T2 cells. Peptide dependent increase of HLA-A^*^0201 expression measured by flow cytometry is shown (Supplementary Figure 1). Specific binding was correlated to influenza matrix peptide (GILGFVFTL) binding. Peptide CHM1^319^ and previously published peptide EZH2^666^ (Steele *et al*, 2006) demonstrated strong HLA-A^*^0201 binding whereas peptide CHM1^38^ revealed no binding at all in this assay. Peptides CHM1^319^ and EZH2^666^ were chosen for subsequent *in vitro* priming of T cells.

### Selection of peptide- and ET-specific T cells

**Figure 2:**
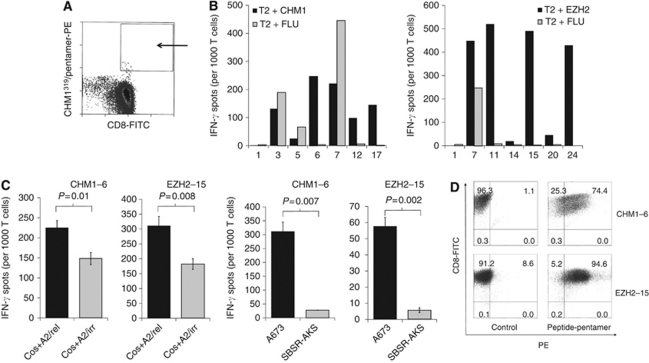
Selection and antigen-specificity of allo-restricted T cells. (**A**) The CD8^+^ Peptide-HLA-A^*^0201-multimer^+^ T cells were sorted by flow cytometry directed cell sorting. An example for CHM1^319^-peptide-specific T cells is given: 1560 cells of 1.5 × 10^7^ input cells were collected in the indicated gate (arrow). *In vitro* primed T cells were pooled and stained with a specific peptide CHM1^319^/HLA-A^*^0201-multimer-PE and counterstained with an anti-human CD8-FITC mAb for cell sorting. Cell sorting was executed on a FACS Aria. (**B**) Sorted T cells were expanded using limiting dilution and screened for antigen specificity in IFN*γ* ELISpot: T2 cells pulsed with CHM1^319^ (VIMPCSWWV), EZH2^666^ (YMCSFLFNL) or as a control FLU (GILGFVFTL) peptide; T-cell lines screened for CHM1 specificity; line 6, 12 and 17 were further expanded (left). T-cell lines screened for EZH2 specificity; lines 11, 15 and 24 passed this screen (right). (**C**) Cos-7 cells were transiently transfected by lipofection with human HLA-A^*^0201 cDNA and expression constructs with the gene of interest or GFP cDNA as an irrelevant control (left). HLA-A^*^0201-specific recognition of ET-cell lines (right). A673 cells are HLA-A^*^0201^+^ expressing the antigen whereas SBSR-AKS is an ET-cell line expressing the target antigen, but is negative for HLA-A^*^0201. IFN-*γ* release was measured in triplicate. Error bars represent s.d. *P*-values <0.05 indicate significant difference (two-tailed *t*-tests were used). (**D**) Flow cytometric determination of peptide specificity of T-cell lines CHM1-6 (top panel) and EZH2-15 (bottom panel) with specific peptide-multimers, irrelevant peptide-multimers served as a control.

Although autologous HLA-A^*^0201 restricted CD8^+^ T cells specific for either EZH2^666^ or CHM1^319^ peptide were easily identified, they were in no case able to recognise HLA-A^*^0201^+^ ET cells (Supplementary Figure 2). Therefore, we focused our attention on the establishment of peptide-specific allo-restricted T cells. For this purpose, *in vitro* generated, mature HLA-A^*^0201^+^ DC were pulsed with either CHM1^319^ or EZH2^666^, which were then used to stimulate purified HLA-A^*^0201^−^ CD8^+^ T cells twice in a 7-day interval (see Materials and Methods). Subsequently, to separate allo-reactive CTL from allo-restricted CTL, peptide/HLA-A^*^0201^+^ multimers were used to label allo-restricted CD8^+^ T cells (Borg *et al*, 2005). The CTL peptide/HLA-A^*^0201^+^ multimer staining was highly specific and usually stained only between 0.1–0.4‰ cells of the stimulated T-cell population. Peptide-multimer-positive T cells were sorted by FACS. Figure 2A provides an example of these marginal T-cell populations that were positive for both peptide/HLA-A^*^0201-multimer and CD8, here specifically stained with the CHM1^319^/multimer. Subsequently, sorted T cells were expanded using limiting dilution and tested for specificity in ELISpot assays.

In a first screen, the expanded T-cell lines were tested for specific IFN-*γ* release against individual peptides: T2 cells were either pulsed with CHM1^319^ or EZH2^666^, or the influenza-derived peptide (GILGFVFTL) as a control. For example, of the T cells initially specifically selected with the CHM1^319^/HLA-A^*^0201-multimer, 96 cell *γ* release against CHM1 lines were grown and tested for specific IFN-^319^ peptide. The results of seven lines are shown in Figure 2B, left. One line that passed this screen (CHM1-6) was further expanded and retested on T2 cells (Supplementary Figure 3, left) as well as Cos-7 cells, which were double-transfected with an HLA-A^*^0201 expression plasmid and a CHM1 cDNA encoding vector, confirming specific recognition and peptide presentation (*P*=0.01, two-tailed *t*-test; Figure 2C, left). Furthermore, subsequent analysis demonstrated correct HLA-A^*^0201-restricted recognition of ET cell lines (*P*=0.007, two-tailed *t*-test; Figure 2C, right). A similar screen for T cells specific for EZH2^666^ peptide identified three lines EZH2-11, -15, and -24 with peptide-specific recognition on T2 cells (Figure 2B, right). One line that was further expanded and repeatedly tested (Supplementary Figure 3, right), revealed specific recognition of processed EZH2^666^ peptide on double-transfected Cos-7 cells (*P*=0.008, two-tailed *t*-test; Figure 2C, left) and HLA-A^*^0201 specific identification of ET lines (*P*=0.002, two-tailed *t*-test; Figure 2C, right). In flow cytometry, these two lines CHM1-6 (specific for CHM1^319^) and EZH2-15 (specific for EZH2^666^) were only positive for V*β* 13.2 (CHM1-6) or V*β* 13.1 (EZH2-15) (data not shown). Both lines stained positive with their respective peptide/HLA-A^*^0201-multimer (Figure 2D) and were CD27^low^, CD28^–^, CD45RA^low^, CD56^+^, CD62L^–^, IL7R^–^, CCR5^–^, and CCR7^–^ (data not shown).

### Allo-restricted T cells mediate Ewing tumour-specific cytotoxicity

**Figure 3:**
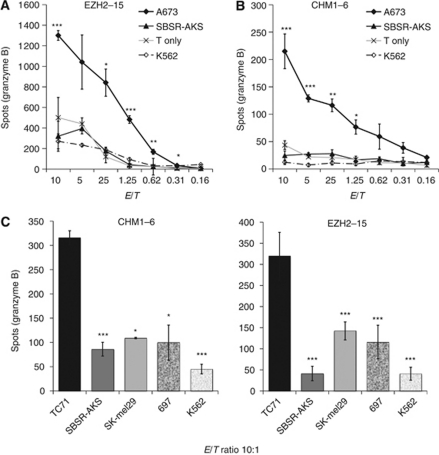
Specific cytotoxicity of allo-restricted T cells directed against HLA-A0201^+^ Ewing tumour cells. Cytotoxicity of allo-restricted T-cell lines was evaluated by target-specific granzyme B release in ELISpot assays. E/T: effector to target ratio. (**A**, **B**) A673: HLA-A^*^0201^+^ ET-cell line; SBSR-AKS: HLA-A^*^0201^–^ ET-cell line; T only: spontaneous release of T-cell lines without target cells; K562: NK-cell control. (**C**) EZH2-specific line EZH2-15 and CHM1-specific line CHM1-6 were retested at a defined E/T ratio against selected cell lines to further evaluate their target specificity. TC-71: HLA-A^*^0201^+^ ET; SK-Mel 29: HLA-A^*^0201^+^ Melanoma; 697: HLA-A^*^0201^+^ paediatric cALL. *P*-values < 0.05 indicate significant difference. Asterisks indicate significance levels of (3A and 3B, Welch two sample *t*-Test) A673 lysis compared with SBSR-AKS lysis or (3C, two-tailed *t*-Test) TC71 lysis compared with respective control cell lines (^*^P<0.05; ^**^P<0.01; ^***^P<0.001).

**Figure 4:**
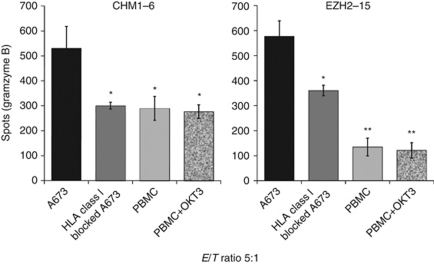
Low granzyme B responses against HLA class I blocked A673, SBSR-AKS and HLA-A^*^0201^+^ PBMC compared with unblocked A673. HLA class I blocking before granzyme B ELISpots caused reversion of specific recognition by CHM1^319^ or EZH2^666^ peptide specific CD8^+^ T cells at an effector to target (E/T) ratio of 5:1. Granzyme B release upon contact with irradiated OKT3-stimulated/unstimulated HLA-A^*^0201^+^ PBMC remained low compared with unblocked A673 at the same E/T ratio. Asterisks indicate significance levels of A673 lysis compared with respective controls (two-tailed *t*-test, ^*^P<0.05; ^**^P<0.01).

To test for ET specific cell-mediated cytotoxicity of allo-restricted T-cell lines, we investigated their ability for antigen-specific granzyme B release in the ELISpot assay (Shafer-Weaver *et al*, 2003; Anderson *et al*, 2007). Both T-cell lines demonstrated a specific granzyme B release only when tested in the appropriate antigen/HLA-A^*^0201-restriction combination, while HLA-A^*^0201^–^ ET cells recognition (SBSR-AKS cells) and possible NK-cell activity, as tested on K562 cells, was not higher than background level of pure T cells (overall *P*<0.05 until effector to target ratio reached 1.25, Welch two sample *t*-test; Figures 3A and B). Retesting at a fixed effector to target ratio of 10 : 1 only identified a significant granzyme B release when these T-cell lines recognised HLA-A^*^0201^+^ ET cells (all *P*<0.05; two-tailed *t*-test; Figure 3C). HLA-restricted recognition was reversed after blocking with an HLA-A^*^0201 blocking antibody. Furthermore, HLA-A^*^0201^+^ PBMC or OKT3 activated, HLA-A^*^0201^+^ T cells where only minimally detected by these allo-restricted T cells, supporting HLA-A^*^0201-restricted antigen-specific cytotoxicity of our selected T-cell lines (all *P*<0.05, two-tailed *t*-test; Figure 4). General feasibility of this approach was further demonstrated by our ability to identify and sufficiently expand several of such T-cell lines derived of five independent donors tested (Table 1).

### ET-specific T cells delay tumour growth in Rag2^−/−^*γ*_C_^−/−^ mice after adoptive transfer

**Figure 5:**
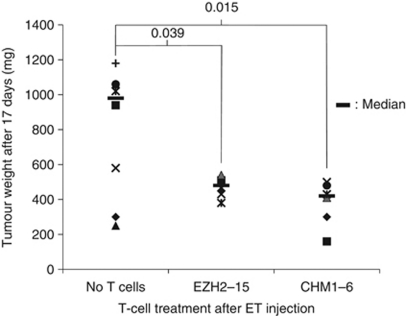
ET-specific T cells delay tumour growth in Rag2^−/−^*γ*_C_^−/−^ mice after adoptive transfer. Rag2^−/−^*γ*_C_^−/−^ mice were challenged s.c. intra-inguinally with 2 × 10^6^ EWS-FLI1^+^ HLA-A^*^0201^+^ A673 ET cells and received 2 × 10^6^ CHM1^319^ or EZH2^666^-specific T cells by i.v. injection 3 days later. Mice were killed and analysed on day 17. Individual mice are represented by symbols. Median tumour weights are indicated by black bars. A *P*-value <0.05 indicates a significant difference between tumour weights of mice treated with EZH2-15 or CHM1-6 compared with controls (Welch two sample *t*-test).

To analyse whether such allo-restricted cytotoxic T cells can inhibit tumour growth *in vivo*, we challenged Rag2^−/−^*γ*_C_^−/−^ mice s.c. intra-inguinally with EWS-FLI1^+^ HLA-A^*^0201^+^ A673 ET cells, followed by i.v. injection of EZH2 (*n*=7) or CHM1 (*n*=6) specific T cells 3 days later (see Materials and Methods). Control mice (*n*=8) did not receive T-cell treatment. Median tumour weights of mice receiving T cells were significantly lower compared with control mice (*P*=0.015 for EZH2- and 0.039 for CHM1 study group, respectively, compared with controls, Welch two sample *t*-test; Figure 5). None of the treated mice showed any signs of GvHD upon analysis.

## Discussion

Ewing tumour are highly malignant tumours of neuroectodermal or endothelial origin (Schmidt *et al*, 1985; Staege *et al*, 2004) and are molecularly defined by ews/ets translocations. In all, 85% of ET are characterized by a specific EWS-FLI1 translocation fusing the gene coding for the ribosomal binding protein EWS to the gene coding for the transcription factor FLI1. The resulting chimeric transcription factor has been implicated in tumour genesis and is tumour-specific (Kovar, 1998; de Alava and Gerald, 2000). However, despite an MHC class II restricted peptide derived from the fusion region of EWS-FLI1 that is able to initiate a CD4^+^ T-cell response (Meyer-Wentrup *et al*, 2005), no immunogenic ET-specific MHC class I binding peptide derived from this fusion region has been identified yet. To further determine possible ET-specific immunogenic peptides, we utilised high-density DNA microarrays for the identification of ET-specific gene expression profiles in comparison with 133 normal tissues of diverse origin (normal body atlas, NBA) and identified 37 genes that were highly upregulated or specifically expressed in ET (Staege *et al*, 2004). Of these, CHM1 and EZH2 revealed specific or at least strong overexpression in ET.

Chondromodulin-I is a glycoprotein that is normally expressed mainly in immature cartilage, stimulating proteoglycan and DNA synthesis, proliferation and differentiation of chondrocytes. It inhibits angiogenesis *in vitro* and *in vivo* (Hiraki *et al*, 1997, 1999). The overexpression of such a molecule in a malignant tumour is surprising, but may be associated with the reduced microvessel density in ET and the observation that an increased aggressiveness of hypoxic tumour cells may correlate with increased metastasis and inferior prognosis (Dunst *et al*, 2001). Chondromodulin-I was previously not known to be tumour-associated.

Enhancer of Zeste, Drosophila Homolog 2 is part of the polycomb repressor complex 2 (PRC2) and within this complex it silences target genes by methylating lysine 27 on histone 3 (H3K27). Enhancer of Zeste, Drosophila Homolog 2 is already active at gastrulation (Sparmann and van Lohuizen, 2006). We found EWS-FLI1 to be bound to the EZH2 promoter *in vivo*, inducing EZH2 expression in ET and mesenchymal stem cells. Downregulation of EZH2 by RNA interference suppressed ET tumour development and metastasis in immunodeficient Rag2^−/−^*γ*_C_^−/−^ mice. Enhancer of Zeste, Drosophila Homolog 2 maintained an undifferentiated stemness phenotype in ET (Richter *et al*, 2009), implicating that EZH2 might have a central role in ET pathology (Burdach *et al*, 2009). Enhancer of Zeste, Drosophila Homolog 2 upregulation is known to be associated with poor prognosis in prostate cancer (Varambally *et al*, 2002). As polycomb group proteins are known to be vitally involved in transcriptional control and carcinogenesis in several human tumours (Simon and Lange, 2008), EZH2 may be less susceptible to the development of immune escape variants. Peptide EZH2^666^ was already validated as a target for cancer immunotherapy (Steele *et al*, 2006).

Mixed results have been observed with autologous SCT for patients with high risk or recurrent ET. Whereas some studies reported improved disease free survival over historical controls (Burdach *et al*, 1991, 2003; Paulussen *et al*, 1998; Burdach, 2004), others observed no long-term benefit compared with conventional therapies (Cotterill *et al*, 2000; Meyers *et al*, 2001). These findings emphasise the need for alternative approaches. In ET patients with vast bone affection and poor prognosis, allogeneic SCT is a therapy option (Burdach *et al*, 2000; Koscielniak *et al*, 2005; Lucas *et al*, 2008). However, the desired GvT effect is intrinsically tied to an often-pronounced GvHD, mediated by allo-reactive T cells. To specifically direct such T cells against the tumour, it is necessary to identify the allo-restricted tumour-specific T cells within an allogeneic T-cell population (Dutoit *et al*, 2002; Mutis *et al*, 2002; Amrolia *et al*, 2003; Whitelegg *et al*, 2005; Schuster *et al*, 2007).

A recent retrospective study based on data drawn from the EBMT-, PRST-, APBMT-, and MetaEICESS-registries revealed that there is no improvement of survival of ET patients receiving reduced intensity conditioning compared with high-dose conditioning before allogeneic stem cell transplantation with HLA-matched grafts, implicating absence of a clinically relevant graft *vs* ET effect (Thiel *et al*, 2011). Reduced intensity conditioning regimen followed by haploidentical stem cell transplantation is subject to various ongoing prospective trials and may increasingly replace HLA-matched approaches. Thus, HLA-A^*^0201^+^ ET patients may profit from a treatment based on adoptive transfer from ET-specific T cells of an HLA-A^*^0201^−^ donor after haploidentical stem cell transplantation.

We isolated allo-restricted T cells by MHC multimer-staining and cell sorting. Using this technique, we have succeeded in establishing T-cell lines directed against several HLA-A^*^0201-restricted peptides derived from ET-specific antigens. Reliable *in silico* prediction algorithms are helpful tools to identify a CTL epitope (Larsen *et al*, 2005). Still, *in silico* high scoring epitope candidates have to be confirmed for binding to HLA-A^*^0201. We not only verified the already published EZH2^666^ peptide as a binding peptide on T2 cells (Steele *et al*, 2006), but identified CHM^319^ as a new good binding peptide (Supplementary Figure 1). As CHM1^319^ had been a previously undescribed peptide, it could have been possible that it represented an artificial epitope. Therefore, the simian cell line Cos-7 was co-transfected with vectors containing the human HLA-A^*^0201 gene and the gene of interest. Again, not only the EZH2^666^ peptide-specific T cells recognised such double-transfected Cos-7 cells, but also the CHM^319^ peptide-specific T cells specifically released IFN−*γ* when contacting Cos-7 co-transfected cells, indicating processivity of these peptide epitopes. Even though EZH2 is expressed at a low level on a variety of tissues compared with CHM1, it may nevertheless constitute an appropriate target for T-cell therapy after successful engraftment, because of its particularly high expression in ET. The risk of GvHD caused by EZH2^666^-specific T cells is likely to be lower than the risk associated with infusion of blunt donor lymphocytes. Nevertheless, CHM1 represents a more appropriate target and further ET-specific targets remain to be identified and tested.

The T cells isolated here not only specifically recognised peptide-pulsed or antigen-transfected cells in the context of HLA-A^*^0201, but also released granzyme B when recognising HLA-A^*^0201^+^ ET expressing the antigen, while other HLA-A^*^0201^+^ tumour lines and HLA-A^*^0201 negative ET were not affected. Furthermore, efficacy of allo-restricted EZH2^666^ and/or CHM1^319^ specific T cells were confirmed in a xenograft mouse model, where ET growth was significantly delayed after adoptive transfer of such T cells compared with controls and GvHD was absent.

Although we could demonstrate the general feasibility of our approach, with which we were able to generate allo-restricted ET-specific T cells in sufficient numbers of every donor tested, long-term persistence of our T cells *in vivo* has not been analyzed, but may be further investigated in a humanised mouse model (Traggiai *et al*, 2004). Future approaches generating ET-specific T cells against EZH2^666^ and/or CHM1^319^ with a central memory (CM) phenotype (Berger *et al*, 2008) in addition may yield improved anti-tumour efficacy. Furthermore, TCR identification, cloning and transfection into donor CM CD8^+^ T cells before adoptive transfer may constitute an appropriate tool to simplify the generation procedure to obtain ET-specific T cells.

However, the generation of highly specific and efficacious allo-restricted T cells here already yet opens the avenue for new therapeutic strategies in allogeneic stem cell and effector-cell transplantation in the treatment of ET patients.

## Figures and Tables

**Table 1:** CHM1 and EZH2-specific T-cell line data from five different donors

**Donor no.**	**Peptide**	**Sorted cells**	**Tested lines**	**Best specified T-cell lines**	**T2+rel/irr peptide mean of IFN*γ***	**A673/SBSR-AKS mean of IFN*γ* or GB spots**	**Expansion factor after 14 days**
1	CHM1-319	1560	96	2^a^	19.6 and 145	30.7 (IFN*γ*)	80–100
2	EZH2-666	5418	96	4^a^	1.8, 57.8, 490 and 15	10.1 (IFN*γ*)	100–140
3	CHM1-319	590	48	1	5	4.9 (GB)	17
4	CHM1-319	706	9	2	1.6 and 2.3	3.6 and 6.8 (IFN*γ*)	22–24
5	CHM1-319	2160	48	1	61.5	11.1 (IFN*γ*)	50–80

Abbreviations: IFN*γ*=interferon-*γ*; irr=irrelevant; GB=granzyme B; rel=relevant.

a Only one cell line was further tested for A673 and SBSR-AKS discrimination.

CD8^+^ T Cells (6 × 10^6^ to 1 × 10^8^) from five different HLA-A^*^0201^−^ healthy donors were stained and screened for the presence of CHM1^319^ or EZH2^666^ peptide-specific CD8^+^ T cells after priming with peptide-loaded HLA-A^*^0201^+^ dendritic cells. Expanded T-cell lines were tested for specificity in IFN*γ* (and granzyme B for donor no. 3) ELISpot assays using T2 cells pulsed with either relevant or irrelevant peptides and A673 and SBSR-AKS Ewing tumour cell lines as targets with an ratio of 1000 T cells/20 000 target cells (or 200 000 T cells/20 000 target cells in granzyme B assays). Numbers, specificity data and expansion rates are given.
