# Specific recognition and inhibition of Ewing tumour growth by antigen-specific allo-restricted cytotoxic T cells

**DOI:** 10.1038/bjc.2011.305

**Published:** 2011-08-09

**Authors:** U Thiel, S Pirson, C Müller-Spahn, H Conrad, D H Busch, H Bernhard, S Burdach, G H S Richter

**Correction to**: *British Journal of Cancer* (2011) **104**, 948–956; doi:10.1038/bjc.2011.54

When published originally, earlier this year in Volume 104, the authors noticed a couple of errors in the Results section.

The first is in the subheading entitled ‘Selection of peptide- and ET-specific T cells’. In the second paragraph of this subsection, on page 951, the second sentence should read ‘For example, of the T cells initially specifically selected with the CHM1^319^/HLA-A^*^0201-multimer, 96 cell lines were grown and tested for specific IFN-γ release against CHM1^319^ peptide.’

The legend of Figure 4 should read ‘Low granzyme B responses against HLA class I blocked A673 and HLA-A^*^0201^+^ PBMC compared with unblocked A673.’

The publishers and authors are now happy to correct these errors.

## Figures and Tables

**Figure 4:**
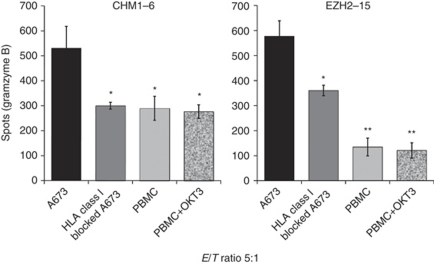
Low granzyme B responses against HLA class I blocked A673 and HLA-A^*^0201^+^ PBMC compared with unblocked A673. HLA class I blocking before granzyme B ELISpots caused reversion of specific recognition by CHM1^319^ or EZH2^666^ peptide specific CD8^+^ T cells at an effector to target (E/T) ratio of 5:1. Granzyme B release upon contact with irradiated OKT3-stimulated/unstimulated HLA-A^*^0201^+^ PBMC remained low compared with unblocked A673 at the same E/T ratio. Asterisks indicate significance levels of A673 lysis compared with respective controls (two-tailed *t*-test, ^*^P<0.05; ^**^P<0.01).

