# Supplementary figures and images for: Specific recognition and inhibition of Ewing tumour growth by antigen-specific allo-restricted cytotoxic T cells
Source: Br J Cancer. 2011 Mar 15;104(6):948–56. doi: 10.1038/bjc.2011.54 (PMC3065285; doi:10.1038/bjc.2011.54)

## Slide 1
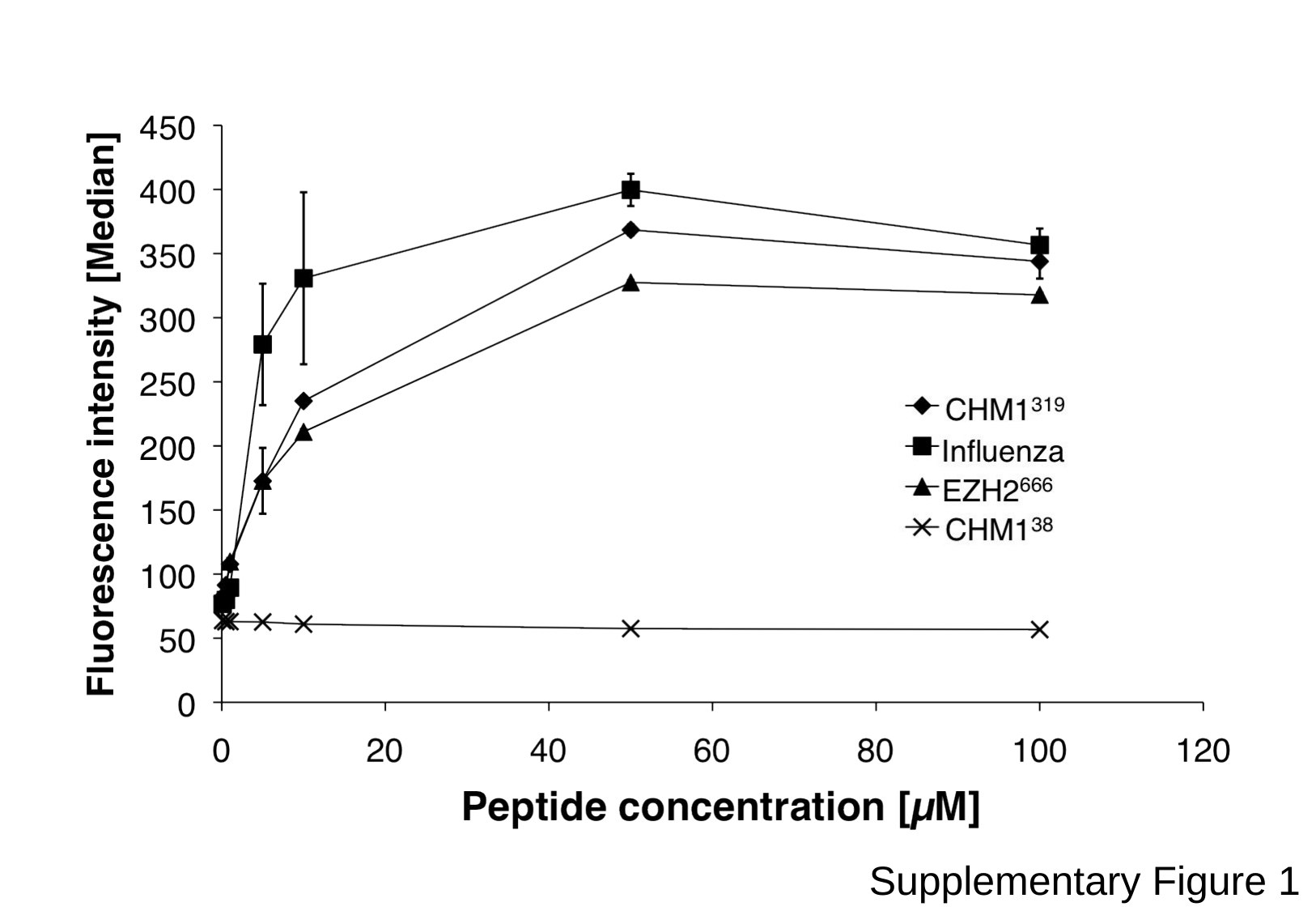

Supplementary Figure 1

## Slide 2
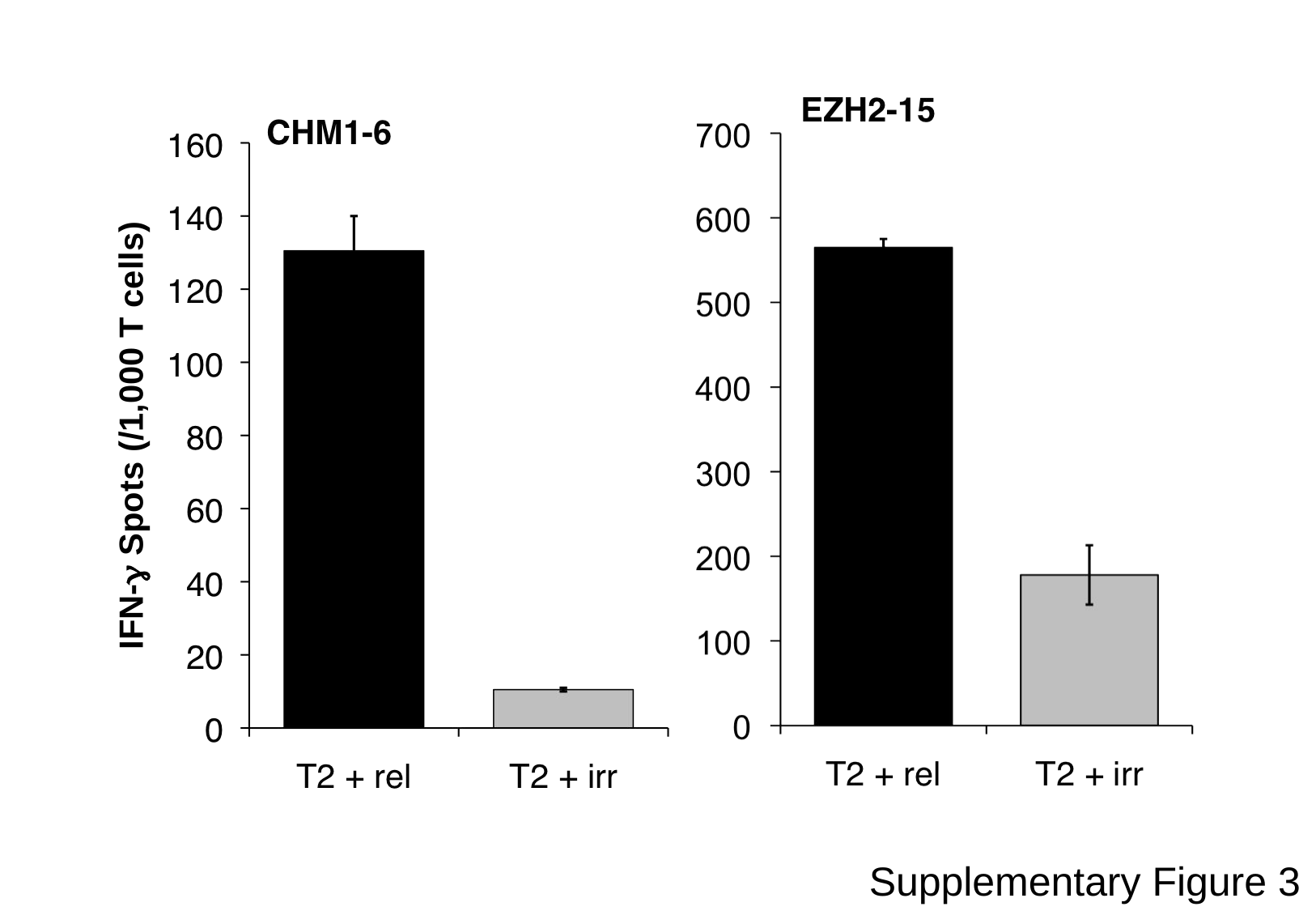

IFN-γ Spots (/1,000 T cells)
Supplementary Figure 3

Supplement: Supplementary Figures 1 and 3 [file bjc201154x1.ppt]

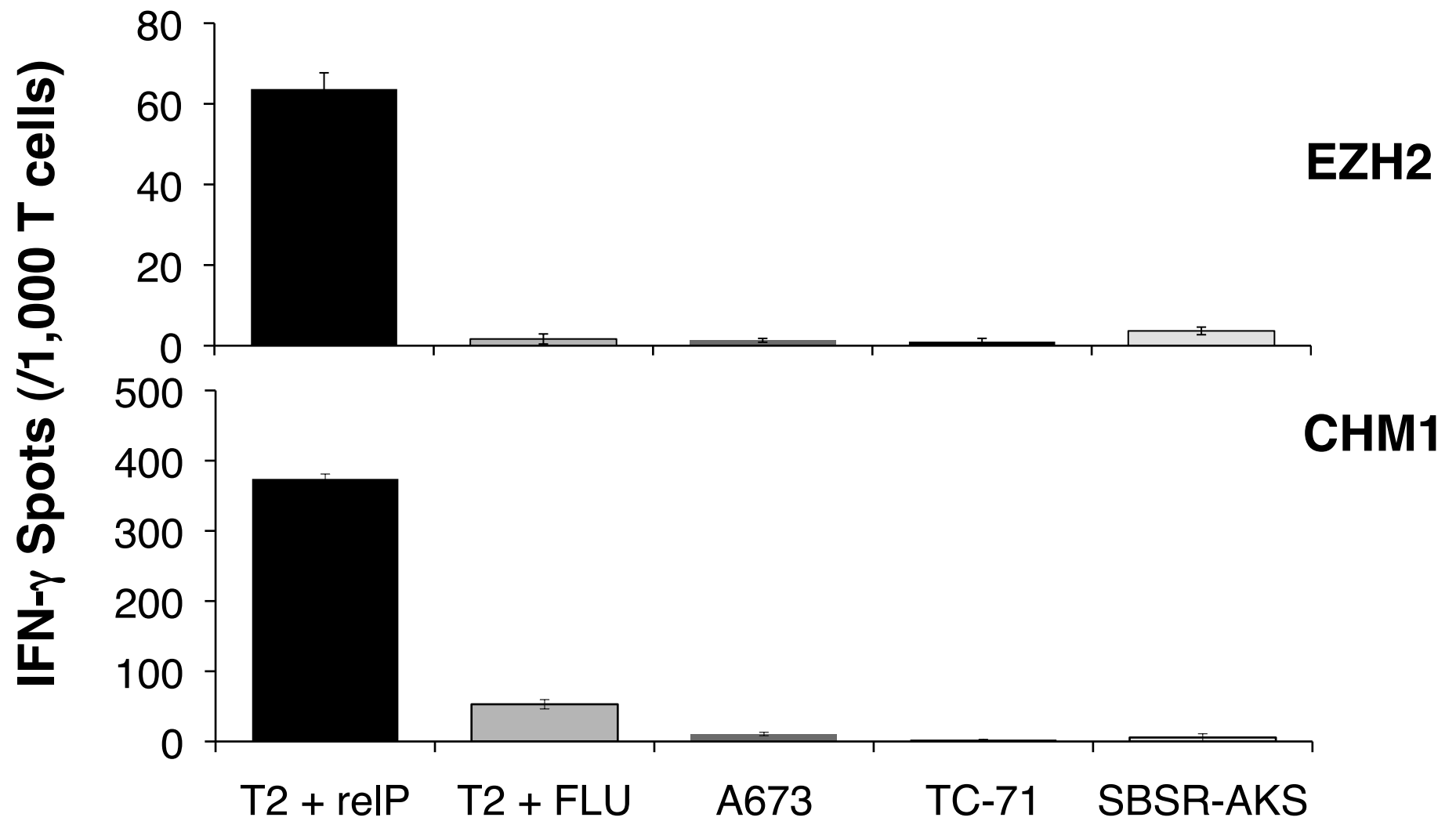

Supplementary Figure 2

Supplement: Supplementary Figure 2 [file bjc201154x2.pdf]
