# Supplementary material for: Specific recognition and inhibition of Ewing tumour growth by antigen-specific allo-restricted cytotoxic T cells
Source: Br J Cancer. 2011 Mar 15;104(6):948–56. doi: 10.1038/bjc.2011.54 (PMC3065285; doi:10.1038/bjc.2011.54)
Supplement: Supplementary Information [file bjc201154x3.doc]

**Supplemental Information**

***In silico* Prediction of Peptide Epitopes and HLA-A*0201/peptide Binding Assay**

Immunogenic peptide epitopes derived from ET specific target genes with high score binding to HLA-A*0201 were *in silico* predicted with help of several web-based methods comprising: SYFPEITHI: http://www.syfpeithi.de/Scripts/MHCServer.dll/EpitopePrediction.htm (Rammens*ee et* al, 1999), BIMAS: http://www-bimas.cit.nih.gov/molbio/hla_bind/, (Park*er et* al, 1994) algorithms and NetCTL: http://www.cbs.dtu.dk/services/NetCTL/ to include proteasomal cleavage prediction. The best scoring peptide candidates were synthesized by Thermo Fisher Scientific (Ulm, Germany) and further subjected to HLA-A*0201-peptide binding *in vitro* to T2 cells: Cells were washed, counted and resuspended in T cell medium (TCM) AIM-V (Life Technologies) supplemented with 5 % human AB serum, 2 mM L-glutamine and 50 µg / ml gentamycine to a concentration of 1 x 10^6^ / ml. For every peptide concentration to be tested T2 cells were pulsed with a serial dilution of 0.1 – 100 µM peptide and then incubated for 16 hours at 37 ^0^C and 5% CO_2_. Subsequently, cells were washed three times in PBS / 2% FCS and stained with an anti-HLA-A*0201-FITC antibody (BB7.2, BD Biosciences) to determine increase in HLA expression by flow cytometry on a FACS Calibur (BD Biosciences). Influenza peptide (GILGFVFTL, (Nijm*an et* al, 1993) served as a positive control, cells without peptide as a negative control.

**Transfection by Lipofectamine**

The day before transfection Cos-7 cells were plated into 6 well plates at a concentration resulting in 80 – 90 % confluency the following day of transfection. 3 μg of DNA were diluted with 250 µl OptiMEM (Life Technologies) and mixed gently. In another tube 7.5 μl Lipofectamine (a 3 : 1 (w / w) liposome formulation of the polycationic lipid 2,3-dioleyloxy-N-[2(sperminecarboxamido)ethyl]-N,Ndimethyl-1-propanaminium-trifluoroacetate (DOSPA) and the neutral lipiddioleoyl phosphatidylethanolamine (DOPE) in membrane-filtered water; Life Technologies) was diluted with 250 μl OptiMEM and incubated for 5 minutes at RT. Diluted DNA and Lipofectamine reagent were combined and incubated for 20 min at RT to allow DNA-liposome complexes to form. While complexes were forming, cell culture medium was replaced with medium without antibiotics. For each well 500 µl medium containing the complexes was added drop wise onto the cells and incubated at 37 ^0^C in a CO_2_ incubator. 24 hours after transfection efficiency of gene transfer was evaluated.

**Real-time RT-PCR.**

Differential gene expression of cDNA or siRNA transfectants was verified by real-time RT-PCR. Total RNA was reverse transcribed using the Superscript First-Strand Synthesis System with oligo-dT primers (Invitrogen; Karlsruhe, Germany) according to the manufacturer’s instructions. Quantitative real-time PCR was performed by use of TaqMan^TM^ Universal PCR Master Mix (Applied Biosystems, Darmstadt, Germany) and fluorescence detection with an AB 7300 Real-Time PCR System (Applied Biosystems). Gene specific primers and probes were obtained as TaqMan^TM^ Gene Expression Assay sets from Applied Biosystems which consisted of a FAM™ dye-labeled TaqMan^TM^ MGB probe and two unlabeled PCR primers. 20 x stock solutions of these reagents were added to the TaqMan^TM^ Universal PCR Master Mix with cDNA at a final volume of 25 µl. The final concentration of primers and probe were 900 and 250 nM respectively. Inventoried TaqMan^TM^ Gene Expression Assays were used for the genes EZH2 (Hs00544830_m1) and CHM1 (Hs00170877_m1). Gene expression was normalized to GAPDH (Hs99999905_m1).

**References:**

Nijman HW, Houbiers JG, Vierboom MP, van der Burg SH, Drijfhout JW, D'Amaro J, Kenemans P, Melief CJ, Kast WM (1993) Identification of peptide sequences that potentially trigger HLA-A2.1-restricted cytotoxic T lymphocytes. *Eur J Immunol* **23**(6)**:** 1215-9

Parker KC, Bednarek MA, Coligan JE (1994) Scheme for ranking potential HLA-A2 binding peptides based on independent binding of individual peptide side-chains. *J Immunol* **152**(1)**:** 163-75

Rammensee H, Bachmann J, Emmerich NP, Bachor OA, Stevanovic S (1999) SYFPEITHI: database for MHC ligands and peptide motifs. *Immunogenetics* **50**(3-4)**:** 213-9

**Supplemental Figure 1. HLA-A*0201-restricted peptide binding to Tap deficient T2 cells.** T2 cells were pulsed with a serial dilution of 0.1 – 100 µM peptide and then incubated for 16 hours at 37 ^0^C and 5 % CO_2_. Subsequently, cells were stained with an anti-HLA-A*0201-FITC antibody to determine increase in HLA expression by flow cytometry. Peptide concentration dependent increase of HLA-A*0201 expression after CHM1^319^ (VIMPCSWWV) and EZH2^666^ (YMCSFLFNL) peptide binding are shown. Influenza peptide, FLU (GILGFVFTL) served as a positive control, cells without peptide as a negative control (non-binding peptide). Results of a representative experiment are shown. Error bars represent the standard deviation of the experiment run in duplicate.

**Supplemental Figure 2.** **Antigen-specificity of selected T cell lines after autologeous *in vitro* priming**. The generated T cell lines specifically recognized either CHM1^319^ (top) or EZH2^666^ peptide (bottom), shown as reactivity to the relevant peptide (relP). Influenza peptide (FLU) served as a control. But both T cell lines were unable to recognize ET cell line irrespective of HLA-restriction in IFN-γ ELISpot assay. Representative results of several independent experiments are shown.

**Supplemental Figure 3. Peptide specificity of allorestricted T cell lines CHM1-6 and EZH2-15.** Reactivity was measured by IFN-γ release after recognition of peptide pulsed T2 cells in ELISpot assay. rel: relevant peptide; irr: irrelevant peptide.
